# Administration of Heme Arginate Ameliorates Murine Type 2 Diabetes Independently of Heme Oxygenase Activity

**DOI:** 10.1371/journal.pone.0078209

**Published:** 2013-10-30

**Authors:** Abhijeet K. Choudhary, Jillian Rennie, Carolynn Cairns, Gary Borthwick, Jeremy Hughes, Nicholas M. Morton, David Kluth, Bryan R. Conway

**Affiliations:** 1 Medical Research Council Centre for Inflammation Research, Queens Medical Research Institute, University of Edinburgh, Edinburgh, United Kingdom; 2 University of Edinburgh/British Heart Foundation Centre for Cardiovascular Science, Queens Medical Research Institute, University of Edinburgh, Edinburgh, United Kingdom; Fundação Oswaldo Cruz, Brazil

## Abstract

Amelioration of rodent type 2 diabetes by hemin has been linked to increased heme oxygenase (HO) activity, however alternative mechanisms have recently been proposed for its anti-diabetic effect. We sought to determine the anti-diabetic efficacy of heme arginate (HA), a clinically licensed preparation of heme, and whether its predominant mode of action is via increased HO activity. Intravenous administration of HA reduced hyperglycemia in diabetic (db/db) mice. Co-administration of the HO inhibitor stannous (IV) mesoporphyrin IX dichloride (SM) resulted unexpectedly in a further improvement in glycaemic control despite restoring HO activity to baseline levels. The anti-diabetic effects of HA±SM were associated with increased adiposity, increased serum adiponectin levels, reduced adipose tissue and islet inflammation and preservation of islet β-cell function. HO activity independent effects of HA on adipogenesis and β-cell inflammation were further confirmed in cell culture models using the 3T3-L1 pre-adipocyte and MIN6 β-cell lines, respectively. In conclusion, our work demonstrates that the heme component of HA ameliorates experimental type 2 diabetes by promoting metabolically favourable adipogenesis and preserving islet β-cell function, but this is not mediated via increased HO activity.

## Introduction

Heme is a key endogenous regulator of circadian rhythms and may in addition influence metabolic pathways including gluconeogenesis [1] and adipogenesis [2]. Administration of heme, in the form of repeated intraperitoneal injections of hemin ameliorates experimental type 2 diabetes by reducing systemic inflammation, augmenting β-cell insulin secretion, improving peripheral insulin sensitisation, increasing serum adiponectin levels and by activating skeletal muscle 5′ adenosine monophosphate-activated protein kinase (AMPK) [3]–[5].

The anti-diabetic effect of heme was linked to activation of the heme oxygenase (HO) system [3]. This is comprised of the inducible (HO-1) and constitutive (HO-2) isoforms and constitutes the principal pathway of heme degradation, resulting in generation of iron, carbon monoxide and biliverdin, all of which exhibit cytoprotective and anti-inflammatory properties [6]. Ndisang and Jadhav have demonstrated that administration of hemin up-regulates HO activity in non-obese insulin-resistant Goto-Kakizaki rats via induction of HO-1 and that the anti-diabetic effect of heme is abrogated by concurrent administration of an inhibitor of HO activity, chromium (III) mesoporphyrin IX chloride (CrMP) [3]. In addition, cobalt (III) protoporphyrin IX chloride (CoPP), an alternative inducer of HO activity, ameliorates insulin resistance in obese (ob/ob) mice in a HO activity-dependent manner [7].

However, administration of heme may have pleiotrophic effects on metabolism that are independent of HO activity. For example, binding of heme to the nuclear hormone receptor, Rev-erb-α, recruits nuclear co-repressor-histone deacetylase 3 complexes (NCoR–HDAC3) to the promoters of gluconeogenic genes, thereby inhibiting gluconeogenic gene expression and glucose output in hepatocytes *in vitro* [1]. In addition, by acting as a Rev-erb-α ligand, heme promotes adipogenesis *in vitro* in an analogous manner to peroxisome proliferator activator receptor-γ (PPAR-γ) agonists [2]. Notably, administration of synthetic Rev-erb agonists ameliorates glucose intolerance in diet-induced obesity [8].

Heme arginate (HA), in which heme is complexed with L-arginine (LA) to improve the solubility and stability of the heme component [9], is licensed for intravenous use in humans in the treatment of acute porphyria [10], and may therefore be readily be taken forward in clinical trials in type 2 diabetes. Prior to clinical use we wished to confirm that HA reduced hyperglycaemia in the leptin-receptor deficient (db/db) model of type 2 diabetes. In addition, we wished to confirm the HO activity-dependence of this effect by concurrently administering stannous (IV) mesoporphyrin IX dichloride (SM), an inhibitor of HO activity. Our results support an anti-diabetic effect for HA, however they challenge the concept established from previous work that heme is predominantly acting via induction of HO activity.

## Methods

### Preparation of Porphyrin-based Chemicals

HA was purchased from Orphan Europe (Paris La Défense, France) and diluted to 5 mg/ml in sterile phosphate-buffered saline (PBS) prior to use *in vivo*. The HO-1 activity inhibitors, stannous (IV) mesoporphyrin IX dichloride (SM), and chromium (III) mesoporphyrin IX chloride (CrMP) and an alternative HO-1 inducer, cobalt (III) protoporphyrin IX chloride (CoPP) were purchased from Frontier Scientific (Logan, U.S.A.). SM was diluted to a concentration of 5 mg/ml in 10 mmol/l Tris-NaOH solution pH 12.0, and adjusted to pH 7.4 using 0.1N hydrochloric acid prior to *in vivo* use. For the *in vitro* experiments SM, CrMP and CoPP were prepared at a final concentration of 10–20 mM, pH 7.4 and aliquots were stored at 4°C for 2 weeks prior to use. All of the porphyrin-based compounds were prepared under minimal light. All other chemicals were from Sigma Aldrich (Dorset, U.K.) unless stated otherwise.

### *In vivo* Study Design and Functional Tests

The research was performed under a UK Home Office Project licence number (60/4002) and approved by the Ethical Review Committee of the University of Edinburgh. 10 week old male leptin receptor-deficient db/db mice (Harlan labs, Blackthorn, U.K.) were allotted into groups matched for baseline weight. A pilot study comprised three groups of db/db mice (n = 4/group), which were administered phosphate-buffered saline (PBS), HA (15 mg/kg) or L-arginine (LA, 16 mg/kg) intravenously twice weekly for eight weeks. The dose and frequency of HA administration was based on preliminary studies which demonstrated that 15 mg/kg significantly induced HO activity with no evidence of toxicity and that the induction of HO-1 protein began to diminish by 72 hours following injection (data not shown). The main study included 4 groups of db/db mice which were treated with: PBS (n = 4), HA alone (n = 4), SM alone (n = 5), or HA+SM (n = 8). 15 mg/kg HA (or 150 µl PBS) was administered twice weekly via tail vein injection and 20 mg/kg SM (or 250 µl Tris-HCl solution) was administered intraperitoneally thrice weekly. The dose and frequency of administration of SM was identical to that previously employed to inhibit HO activity in ob/ob mice [7]. Furthermore, in preliminary experiments we confirmed that this dose was sufficient to abrogate the HA-induced increase in HO activity (data not shown). As an additional control, 10 week old lean non-diabetic C57BL/KsJ (lean) mice were treated with 150 µl PBS twice weekly via tail vein.

Body weights were measured weekly. Mice were fasted fortnightly at 0800 hours and blood glucose concentrations were measured in tail-nick blood samples taken 4 hours later using a glucometer (Accu-check Aviva glucometer, Roche, U.S.A.). The mean daily food intake was determined over a 6-day period during week 5–6 of the study. Serum insulin, high molecular weight (HMW) adiponectin, leptin and non-esterified fatty acid (NEFA) concentrations were determined in the serum of mice after a 4 hour fast using the ultra-sensitive mouse insulin ELISA kit (Crystal Chem Inc, Downers Grove, U.S.A.), the adiponectin ELISA kit (Alpco, Salem, U.S.A), the mouse leptin ELISA kit (Chrystal Chem) and the NEFA–HR2 kit (Wako Chemicals, Neuss, Germany) respectively, according to the manufacturer’s guidelines. Homeostatic model assessments (HOMA) for insulin resistance (HOMA–IR) and β-cell function (HOMA-β) were calculated using the formulae: HOMA–IR = (Glucose x Insulin)/22.5 and HOMA-β = (20 x Insulin)/(Glucose –3.5), where the glucose and insulin values are expressed as mmol/l and mU/l respectively.

### Heme Concentration and Heme Oxygenase Activity

Liver lysates were prepared in phosphate buffer (0.1 M di-potassium phosphate, 2 mM magnesium chloride, pH 7.4), the protein concentration was determined using the BCA assay (Thermo scientific, Wilmington, U.K.) and adjusted to 2 mg/ml. The heme content in the liver and cell lysates was determined using the QuantiChrom heme assay kit (BioAssay Systems/Universal Biologicals Ltd, Cambridge, U.K.).

HO activity was determined by the paired enzyme assay in which heme is converted into biliverdin by HO and then into bilirubin by biliverdin reductase in the presence of an NADPH generating system [11]. Briefly, 1 mg of lysate was added to an equal volume of master mix (0.2 U glucose-6-phosphatase dehydrogenase, 4 mM d-glucose-6-phosphate, 100 µM HA, 0.4 nM bilirubin reductase A, 2.5 mM NADPH in phosphate buffer) and incubated in the dark for 1 hour at 37°C in a water bath. The reaction was stopped by addition of chloroform. The bilirubin in the chloroform fraction was measured on a spectrophotometer at *A*_460_–*A*_530_ (ε = 40 mM^−1 ^cm^−1^).

### Western Blotting for HO-1

Tissues including liver and fat were homogenised in ice-cold buffer A (50 mM HEPES, pH 7.0, containing 20 mM NaCl, 1 mM DTT, 10 mM sodium pyrophosphate, 10 mM sodium fluoride, 1 mM sodium orthovanadate and 1x Complete protease inhibitor cocktail) and centrifuged at 10,000 *g* for 15 minutes at 4°C to collect the supernatant and quantify protein content using the BCA assay (Thermo Scientific). Samples of equal protein content were prepared in 5x laemmli sample buffer and heated for 10 minutes at 95°C. Samples containing 30 µg protein and protein standard were resolved on 10% Tris-acrylamide gels at 70 V. The resulting fractionated samples were transferred from the gel onto a Hybond ECL nitrocellulose membrane (Amersham Biosciences, Buckinghamshire, U.K.) at 90 V for 1 hour in transfer buffer (50 mM TRIS, 383.5 mM glycine, 20% (v/v) methanol). The nitrocellulose membrane containing the transferred proteins was blocked in 5% (v/v) non-fat milk solution for 1 hour. Membranes were washed thrice with TBST (50 mM TRIS pH 7.5, 150 mM NaCl, 0.05% (v/v) Tween-20) between incubations. The membranes were incubated overnight at 4°C with primary antibodies for HO-1 (Enzo Life Sciences, Exeter, U.K.) and β-actin (Sigma Aldrich, Dorset, U.K) diluted in 3% milk solution, then incubated with a species appropriate secondary HRP conjugated antibody (Dako, Cambridgeshire, U.K.) diluted in 3% milk solution for 1 hour at room temperature before being treated with ECL western blotting detection reagent (Thermo scientific) and developed.

### Immuno-histochemical (IHC) Staining

We employed 5 µm thick sections of paraffin-embedded, methyl carnoy’s solution-fixed pancreas or fat tissue (serial pancreas sections were cut at least 75 µm apart). The sections were deparaffinised, serially rehydrated and blocked with 3% hydrogen peroxide, Avidin/Biotin blocking kit (Vector laboratories, Peterborough, U.K.) and serum-free protein block (Dako, Cambridgeshire, U.K.) prior to overnight incubation at 4°C with either rat anti-mouse F4/80 (1∶100 dilution; Invitrogen, Paisley, U.K.) or rabbit anti-mouse iNOS (1∶100, BD Transduction laboratory, Oxford, U.K.). Sections were subsequently incubated with a species-specific, biotin-labelled secondary antibody (1∶300, Vector laboratories, Peterborough, U.K.) for 2 hours at room temperature, Vectastain ABC Elite reagent (Vector laboratories, Peterborough, U.K.) for a further 2 hours and developed with diaminobenzide (DAB; Dako, Cambridgeshire, U.K.). For co-staining, the slides were re-blocked and incubated overnight at 4°C with guinea-pig anti-mouse insulin antibody (1∶100, Abcam, Cambridge, U.K.). Sections were subsequently incubated with biotin-labelled goat anti-guinea pig secondary antibody (1∶300, Abcam, Cambridge, U.K.) for 2 hours at room temperature, Vectastain ABC Elite reagent (Vector laboratories, Peterborough, U.K.) for a further 2 hours and stained with HistoGreen chromogen (Linaris biologische produkte, Germany).

### Image-based Quantification

Images of pancreatic islets and adipocytes in epididymal fat pad were captured at 200x magnification on an Axioskop microscope (Zeiss, Hertfordshire, U.K.). The mean islet area, number of F4/80^+^ and iNOS^+^ cells/unit islet area, adipocyte number per x200-power field and adipocyte area were determined using ImageJ software (National Institutes of Health, Bethesda, MD). A minimum of 25 islets/mouse was used for islet-based quantification. For adipose tissue, a minimum of 10 images/mouse with an average of 15 adipocytes/image was used for quantifying adipocyte size and number and percentage area staining F4/80^+^. All scoring was performed in a blinded manner by a single observer.

### *In vitro* Adipogenesis Assay

The murine 3T3 pre-adipocyte cell line (American Type Culture Collection, ATCC) was plated a density of 125,000/well in 12-well collagen-coated plates (BD Biosciences, UK) and cultured in DMEM containing 10% fetal calf serum, 100 U/ml penicillin, 100 µg/ml streptomycin and 2 mM L-glutamine. A standard adipogenic induction cocktail comprising 500 µM 3-isobutyl-1-methylxanthine, 0.25 µM dexamethasone, 1 µg/ml insulin, and 100 nM rosiglitazone was added and after 48 hours the medium was replaced with complete DMEM, containing 1 µg/ml insulin for a further 48 hours, then complete DMEM only for the remaining 6 days. Throughout the differentiation process, the induction cocktail was supplemented with either PBS, 20 µM HA, 20 µM SM, 20 µM CrMP, 20 µM CoPP, 20 µM HA+SM, 20 µM HA+CrMP or 20 µM HA+CoPP. For analysis of gene expression, at days 3, 6 and 10 cells were lysed in 350 µl of RA1 (Macherey-Nagel, Düren, Germany) containing 3.5 µl of β-mercaptoethanol and stored at−80°C for subsequent RNA analysis. For quantification of lipid droplets at day 10, the cells were fixed overnight in 10% formalin before staining with 60% Oil Red solution for 1 hour. The Oil Red was extracted by incubating with a 4% IGEPAL CA-630 solution made up in isopropanol and the absorbance at 550 nm was determined.

### Culture and Differentiation of Stromal Vascular Fraction

Stromal vascular fraction (SVF) cells were extracted from subcutaneous and epididymal adipose tissue from HO-1^+/−^ mice and wild-type (WT) littermates. Adipose tissue was placed in KREB isolation buffer (0.118 M NaCl, 5 mM KCl, 1.2 mM MgSO_4_.7H_2_O, 100 mM NaH_2_PO_4_, 100 mM Na_2_HPO_4,_ 1.2 mM CaCl_2_, 0.1% D-Glucose, 1% BSA) containing 2% type I collagenase (Worthington Biochemical Corporation, NJ, USA ) and digested at 37°C for 20 minutes. The digest was then washed with SVF proliferation culture media (DMEM/HamsF12 1: 1, 10% New-born calf serum, 100 U/ml penicillin, 100 µg/ml streptomycin and 2 mM L-glutamine, 33 µM biotin, 17 µM pantothenic Acid, Invitrogen, Paisley, UK). The upper layer comprising adipocytes was removed and passed sequentially through 250 µM, 100 µM and 40 µM nylon mesh filters (BD Biosciences, Oxford, UK). Single cell suspensions were plated on collagen type 1 coated 6-well plates (BD Biosciences, UK). After 3 days in cell proliferation growth media, cell debris and red blood cells were washed from the SVF culture. Once confluent, the cells were trypsinised and transferred to collagen coated 96-well plates. SVF cells were differentiated in proliferation media containing insulin (5 µg/ml), dexamethasone (100 nM), apotransferrin (10 µg/ml), T3 (200 pM), 3-isobutyl-1-methylxanthine (IBMX, 500 µM) and rosiglitazone (2 µM) with either PBS or HA (20 µM) for 3 days and then for a further 7 days in the same media, but without IBMX or rosiglitazone. Several wells were undifferentiated to serve as controls. After 10 days of differentiation, cells were washed, fixed with 10% formalin overnight, stained with 60% oil red O solution (Sigma, Dorset, UK) and images captured using an inserted Zeiss axioscope.

### Inflammatory Response in MIN-6 β-Cells

Cells from the murine insulinoma MIN6 β-cell line (ATCC) were plated at 500,000/well in 12-well plates for 48 hr in DMEM containing 15% FCS, penicillin (100 U/ml), streptomycin (100 µg/ml) and mercaptoethanol (5 µl/ml). The cells were pre-treated in fresh medium with either PBS or 20 µM SM for 2 hours before adding 20 µM HA. 4 hours later a cytokine mix (CM) containing IL-1β (5 ng/ml), IFN-γ (100 ng/ml) and TNF-α (10 ng/ml) was added to the medium and the cells were incubated for 20 hours and then lysed in 1 ml of TRIZOL reagent and stored at−80°C for subsequent RNA analysis.

### Gene Expression Analysis

Total RNA was extracted from 3T3-L1 cells and from epididymal fat tissues of db/db mice using a NucleoSpin RNA II kit (Macherey-Nagel), or from TRIZOL-lysed MIN6 β-cells. RNA was reverse transcribed to cDNA using the high capacity cDNA reverse transcription kit (Applied Biosystems, Warrington, U.K.). Real-time PCR was performed on a Fast Real time 7500 PCR machine (Applied Biosystems) in triplicate using the following TaqMan inventoried primers (Applied Biosystems): Pref-1 (Mm00839636_g1), PPAR-γ (Mm00839636_g1), FASN (Mm00839636_g1), MCP-1α (Mm00441242), CD68 (Mm00839636_g1), NOS2 (Mm00440502_m1), TNF-α (Mm00443258_m1), IL-1β (Mm00839636_g1), Cxcl-1 (Mm00839636_g1), 18S (Mm00839636_g1) and TATA box binding protein (Mm00839636_g1). The relative expression of target genes relative to invariant control TATA box binding protein (TBP) or 18S was determined using the 2^-ΔΔCt^ formula.

### Statistical Analysis

All data are expressed as mean ± s.e.m. Multiple treatment groups were compared either by one-way analysis of variance or repeated measures two-way ANOVA tests, with Bonferroni’s multiple comparison post-tests, with the significance threshold set at p<0.05. All statistical analysis was performed using GraphPad Prism version 4.0 (GraphPad Software, San Diego, CA, www.graphpad.com).

## Results

### The Heme Component of HA Ameliorates Hyperglycaemia in db/db Mice

An initial pilot experiment confirmed that administration of HA reduced fasting glucose levels and glycosylated haemoglobin concentration (HbA_1C_%) in db/db mice (Table 1). The improvement in glycaemic control occurred despite an increase in body weight and was associated with an increase in fasting serum insulin concentration and hepatic heme oxygenase (HO) activity (Table 1). As HA is a mixture of hemin and LA, an additional control group was injected with LA at an identical concentration to that present in 15 mg/kg of HA. Administration of LA alone failed to replicate the changes in metabolic parameters observed with HA treatment (Table 1).

**Table 1:** Clinical parameters in the pilot study examining the efficacy of heme arginate (HA) and L-arginine (LA) in the db/db mouse model of type 2 diabetes.

Parameters	PBS (n = 4)	LA (n = 4)	HA (n = 4)
Fasting glucose (mmol/l)			
Week 0	18.0±2.0	17.9±2.6	16.8±0.8
Week 8	31.4±0.9	28.8±0.9	16.2±2.5 *** ^†††^
Week 8 HbA_1C_%	8.4±0.4	7.8±1.2	4.6±0.9 *
Body weight (g)			
Week 0	44.8±1.2	43.7±1.0	44.8±1.2
Week 8	47.5±1.7	43.5±2.1	58.0±1.2 * ^††^
Food intake (g/day)	9.1±0.2	10.1±0.3	10.2±0.2
Week 8 Fasting insulin (ng/ ml)	2.4±0.3	2.7±0.3	6.5±1.1 ** ^††^
Hepatic HO activity (pmol bilirubin mg^−1^ protein hr^−1^)	70.0±19.1	90.0±19.1	440.0±14.8 *** ^†††^

Data given are means ± s.e.m. *P<0.05, **P<0.01, ***P<0.001: v PBS. ^††^P<0.01, ^†††^P<0.001: v LA. P-values derived from Bonferroni’s multiple comparison post-test when P<0.05 by one-way analysis of variance. PBS: phosphate buffered saline; HO: heme oxygenase.

### Inhibition of HO Activity Accentuates the Reduction in Blood Glucose and Lipids Observed with HA Therapy

**Figure 1:**
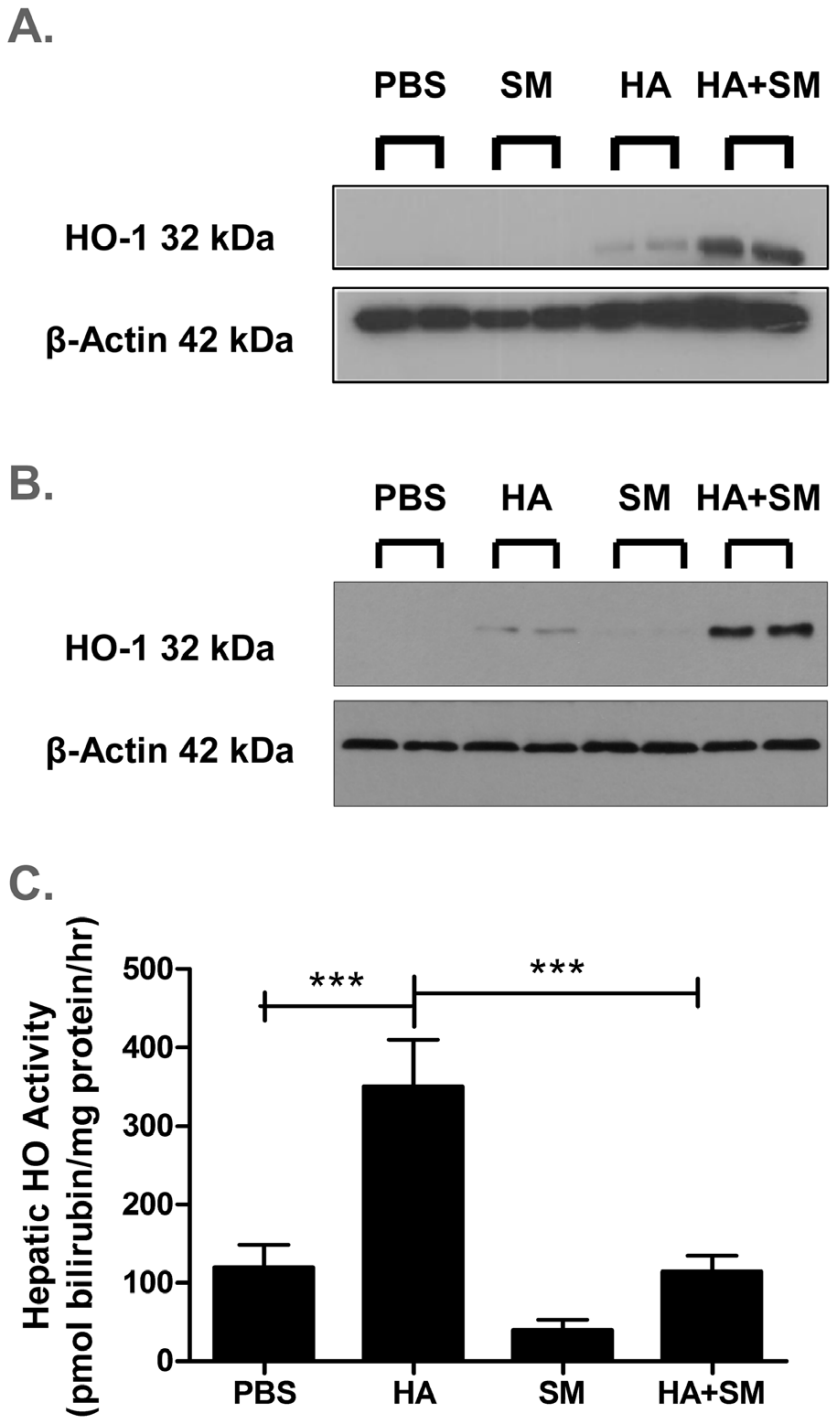
Administration of SM further augmented HO-1 protein expression but abrogated the increase in HO activity induced by HA. Representative immuno-blots of HO-1 expression in **A.** Epididymal fat and **B.** Liver. **C.** Hepatic HO activity in each treatment group. ***P<0.001, P-values derived from Bonferroni’s multiple comparison post-test where P<0.05 by one-way analysis of variance. Data given are means ± s.e.m.

**Figure 2:**
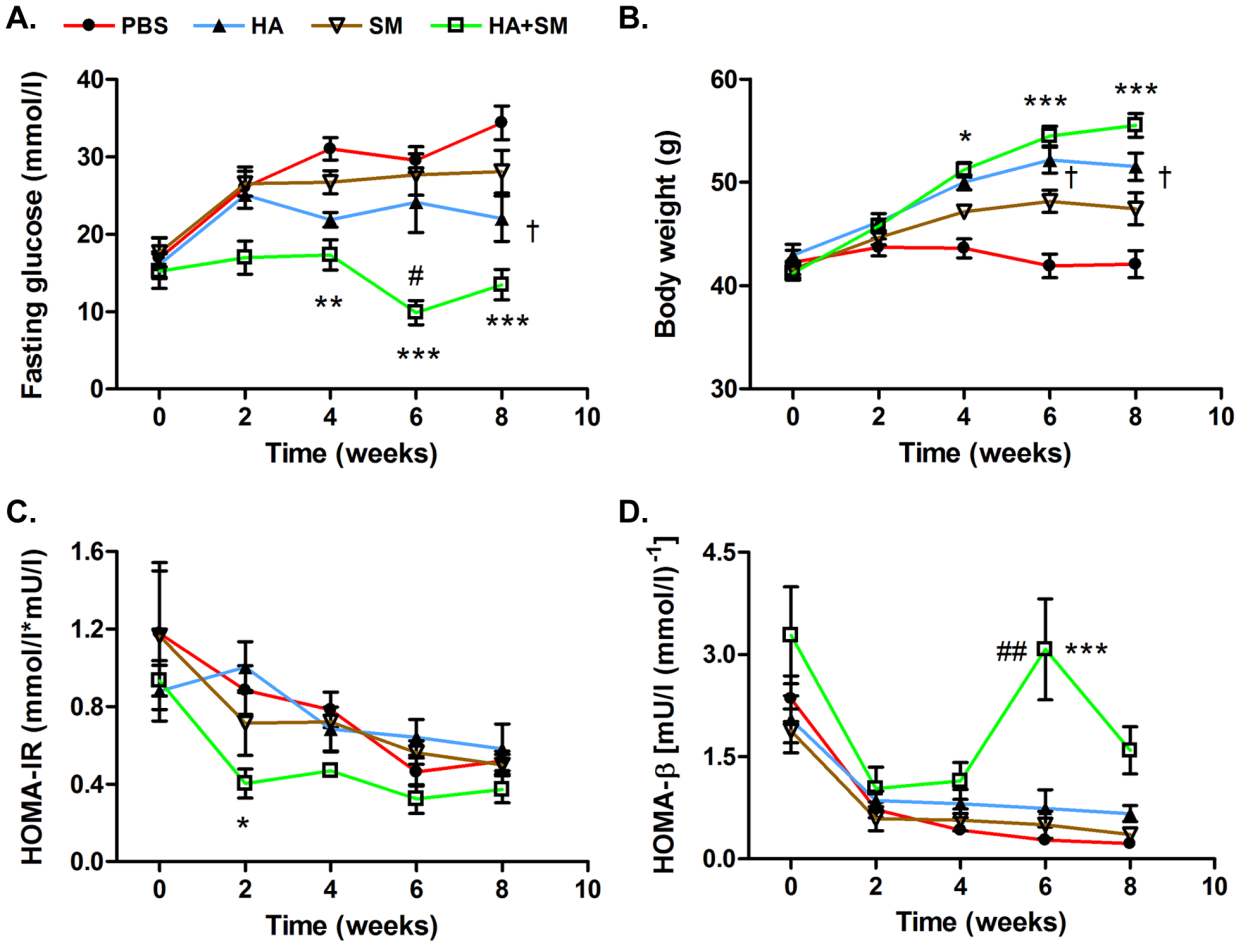
HA±SM improved glycaemic control and β-cell function despite a significant increase in body weight in db/db mice. **A.** Fasting glucose, **B.** Body weight, **C.** HOMA–IR as measure of insulin resistance and **D.** HOMA-β as measure β-cell function during administration of PBS (n = 4), HA (n = 4), SM (n = 5) and HA+SM (n = 8) to db/db mice for 8 weeks. ^†^P<0.05: HA v PBS; *P<0.05, **P<0.01, ***P<0.001: HA+SM v PBS, ^#^P<0.05, ^##^P<0.01: HA+SM v HA. P-values derived by 2-way ANOVA with Bonferroni’s multiple comparison post-test. Data given are means ± s.e.m.

To determine whether the anti-diabetic properties of HA were mediated via an increase in HO activity as previously suggested [3]–[5], db/db mice were treated concurrently with HA and SM, a competitive inhibitor of HO activity. Co-administration of SM led to a further increase in HO-1 protein expression in the adipose tissue and liver (Figure 1 A, B), but completely abrogated the HA-mediated increase in hepatic HO activity (Figure 1C). Despite similar levels of HO activity, db/db mice treated with HA+SM had markedly lower fasting glucose (Figure 2A), HbA_1C_% and non-esterified fatty acid levels (Table 2) compared with PBS-treated db/db mice. These favourable metabolic parameters were observed despite a pronounced increase in body weight in HA+SM-treated animals (Figure 2B). Serial homeostatic model assessments of insulin resistance (HOMA–IR) suggested that the initial reduction in serum glucose in the HA+SM-treated mice was associated with a transient reduction in insulin resistance (Figure 2C). Terminal serum insulin levels were higher in HA+SM-treated mice (Table 2) and serial homeostatic model assessments of β-cell function (HOMA-β) suggested that in the later stages of the experiment HA+SM treatment improved β-cell function compared with PBS-treated db/db mice (Figure 2D).

**Table 2:** Terminal values of clinical parameters in the main study.

Parameters	Lean	db/db			
	PBS	PBS	HA	SM	HA+SM
	(n = 4)	(n = 4)	(n = 4)	(n = 5)	(n = 8)
HbA_1C_%	3.0±0.02	9.0±0.4 ^‡‡‡^	7.8±0.7	8.4±0.7	4.8±0.3 *** ^†††^
Fasting serum insulin (ng/ml)	0.6±0.01	2.0±0.01	3.6±0.5 *	2.4±0.1	3.8±0.5 **
Fasting serum NEFA (mmol/l)	0.6±0.06	1.0±0.09 ^‡^	0.6±0.11	0.7±0.15	0.5±0.03 *
HMW Adiponectin (ng/l)	13.0±1.4	7.3±1.0 ^‡^	8.3±0.5	7.6±0.6	14.1±1.8 ** ^†^
Leptin (ng/l)	1.4±0.2	64.0±3.8 ^‡‡^	72.7±16.2	64.5±9.1	135.8±13.8 ** ^††^
Body weight gain (g)	2.6±0.3	-0.2±1.9	8.6±0.8 **	5.9±1.2 *	14.3±1.4 *** ^†^
Food intake (g/day)	ND	7.9±0.4	7.0±0.7	6.7±0.8	6.1±0.2
Epididymal fat pad weight (g)	ND	1.0±0.01	1.4±0.13 *	1.3±0.03	1.5±0.1 **
Liver weight (g)	ND	3.0±0.1	4.1±0.2	3.8±0.5	4.1±0.3
Hepatic heme concentration(µM mg^−1^ protein)	ND	42±6	71±11	56±11	112±26 *

Lean (C57BL/KsJ) mice were treated with phosphate-buffered saline (PBS) alone and db/db mice were treated with either PBS, heme arginate (HA), stannous (IV) mesoporphyrin IX dichloride (SM) or HA+SM.

Data given are means ± s.e.m. ^‡^P<0.05,^ ‡‡^P<0.01, ^‡‡‡^P<0.001: Lean PBS v db/db PBS; *P<0.05, **P<0.01, ***P<0.001: v db/db PBS. ^†^P<0.05, ^††^P<0.01, ^†††^P<0.001: v db/db HA. P-values derived from Bonferroni’s multiple comparison post-test where P<0.05 by one-way analysis of variance.

### Inhibition of HO Activity Accentuates the Increase in Adiposity Observed with HA Therapy, but Results in a Metabolically Favourable Adipose Tissue Phenotype

**Figure 3:**
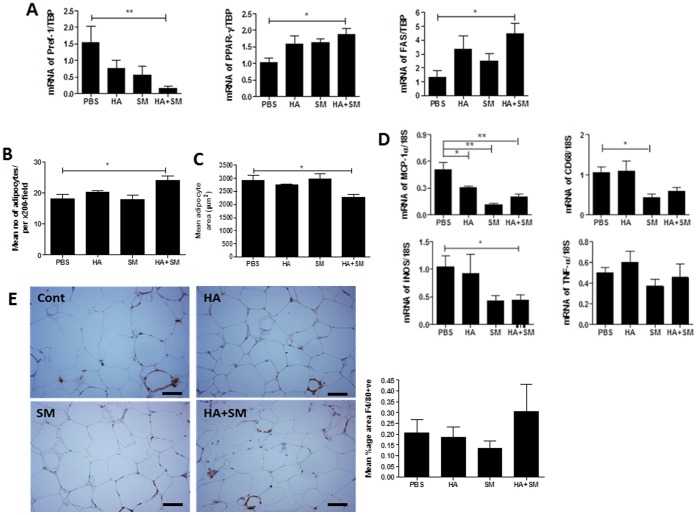
HA+SM promotes adipogenesis and reduces inflammatory gene expression in db/db mice. **A.** Relative expression of Pref-1, PPARγ, FASN genes and **B.** Mean adipocyte number per 200x magnification, **C.** Mean adipocyte area in the epididymal fat pads of each treatment group. **D.** Relative expression of MCP-1α, CD68, iNOS, TNF-α genes, **E.** Representative figures (200x) and quantification for F4/80 staining in adipose tissue. Scale bars represent 25 µM *P<0.05, **P<0.01. P-values derived from Bonferroni’s multiple comparison post-test where P<0.05 by one-way analysis of variance. The data are means ± s.e.m from n = 4–8/group.

Co-administration of SM accentuated the increase in body weight and epididymal fat pad mass (Table 2) observed with HA treatment alone. This occurred despite similar food intake across all treatment groups (Table 2). The increased adiposity was associated with reduced expression of Pref-1 gene but increased expression of the PPARγ and fatty acid synthase (FASN) genes in the epididymal fat pad (Figure 3A), suggesting that HA+SM promoted differentiation of pre-adipocytes into mature adipocytes. Furthermore, the increased in adipose mass was due to hyperplasia rather than hypertrophy of adipocytes as supported by the increased number of adipocytes and reduced mean adipocyte area in HA+SM treated mice (Figure 3 B, C). In keeping with an increase in adiposity, serum leptin levels (Table 2) were increased in db/db mice treated with HA+SM. However, adiponectin levels (Table 2) were restored to the levels observed in lean mice, suggesting the adoption of a more favourable metabolic phenotype. In addition, HA+SM selectively inhibited adipose inflammation, with a reduction in expression of the MCP-1, CD68 and iNOS, but not TNF-α genes (Figure 3D). There was, however no difference between the treatment groups in the mean percentage area of adipose tissue staining for F4/80 (Figure 3E) or in the serum cytokine profile assessed using a cytokine bead array including: TNF-α, interferon-γ, MCP-1, IL-6, IL-10 and IL-12 (data not shown).

### Concurrent Inhibition of HO Activity Potentiates the Ability of HA to Promote Adipogenic Differentiation of pre-adipocytes

**Figure 4:**
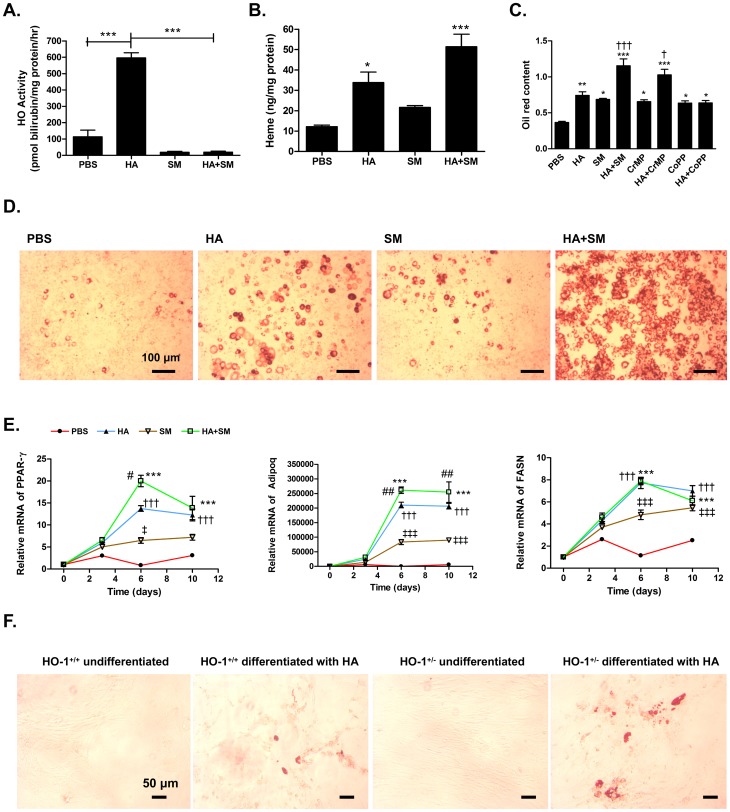
Concomitant administration of SM accentuates adipogenic potential of HA *in vitro*. **A.** HO activity, **B.** Heme concentrations, **C.** Quantification of Oil Red staining and **D.** Representative light microscopic (x100) image of Oil Red uptake in differentiated adipocytes following 10 days treatment of 3T3 cells with an adipogenic differentiation cocktail alone or with the addition of protoporphyrins. B, C: *P<0.05, **P<0.01, ***P<0.001 v PBS; ^†^P<0.05, ^††^P<0.01 v HA. **E.** Time course changes in gene expression of adipogenic genes including PPAR-γ, Adipoq, FASN in 3T3-L1 cells treated with an adipogenic differentiation cocktail alone or with the addition of 20 µM HA, 20 µM SM or HA+SM. ^†††^P<0.001: HA v PBS; ^‡^P<0.05,^ ‡‡‡^P<0.001: SM v PBS, ***P<0.001: HA+SM v PBS; ^#^P<0.05, ^##^P<0.01: HA+SM v HA. P-values derived by 2-way ANOVA with Bonferroni’s multiple comparison post-test. The data are mean ± s.e.m from n = 4/group. F. Representative (200x) images of Oil Red staining of stromal vascular fraction cells from epididymal fat pads of HO-1^+/+^ (left) and HO-1^+/−^ (right) mice either undifferentiated or following differentiation by an adipogenic cocktail in the presence of HA.

The potential of HA±SM treatment to promote adipogenesis was further assessed *in vitro* using the clonal murine preadipocyte 3T3-L1 cell line. Administration of HA markedly increased HO activity in 3T3-L1 cells, and this was completely abrogated by concurrent administration of SM (Figure 4A), resulting in a marked accumulation of heme intracellularly (Figure 4B). While addition of either HA or SM to a standard adipogenic induction cocktail promoted differentiation of 3T3-L1 cells into adipocytes, this was more marked when they were administered in combination (Figure 4 C, D). Adipogenic gene profile assessed by changes in gene expression of PPARγ, adiponectin (Adipoq) and FASN demonstrated that both HA and SM induced adipogenesis during differentiation of 3T3-L1 cells which was augmented when both agents were administered concurrently (Figure 4E). Administration of SM *per se* promoted adipocyte differentiation, therefore to exclude the possibility that this was a specific property of SM; we employed an alternative inhibitor of HO activity, CrMP, which replicated the findings, observed with SM (Figure 4C). Furthermore, administration of cobalt protoporphyrin (CoPP) also promoted adipogenesis (Figure 4C). Finally, to avoid confounding due to non-specific effects of pharmacological HO inhibitors, we assessed the efficacy of HA in differentiating pre-adipocytes from the stromal vascular fraction (SVF) of HO-1 wild-type (WT) and HO-1^+/−^ mice, observing that HA had a more potent adipogenic effect in pre-adipocytes from HO-1^+/−^ mice (Figure 4F).

### Administration of HA and SM in Combination Attenuates Inflammation and Nitro-oxidative Stress in the Islets of db/db Mice

**Figure 5:**
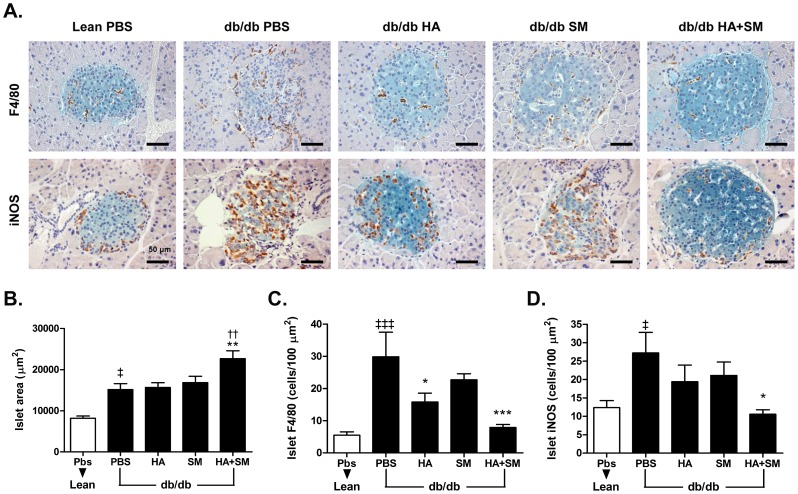
HA+SM increases islet area but reduces islet macrophage infiltration and expression of iNOS protein. **A.** Representative images of the islets from each treatment group. β-cells are visualised by staining with anti-insulin antibody (green), with either F4/80^+^ macrophages (upper panel) or iNOS positive cells (lower panel) stained in brown. Scale bars represent 50 µM. Quantification of **B.** islet cross-sectional surface area; and number of **C.** F4/80^+^ macrophages or **D.** iNOS^+^ cells per unit islet area in each treatment group. ^‡^P<0.05,^ ‡‡‡^P<0.001 v lean PBS, *P<0.05, **P<0.01, ***P<0.001 v db/db PBS, ^††^P<0.01 v db/db HA. P-values derived from Bonferroni’s multiple comparison post-test when P<0.05 by one-way analysis of variance. The data are mean ± s.e.m from n>25 islets/mouse.

The HOMA-β data suggested that concurrent HA+SM therapy may have ameliorated hyperglycaemia in part by preventing the decline in β-cell function observed in PBS-treated db/db mice (Figure 1D). As chronic inflammation [12] and nitro-oxidative stress [13] both promote β-cell dysfunction in type 2 diabetes, we performed immunohistochemistry to assess macrophage (F4/80^+^ cells) infiltration and inducible nitric oxide synthase (iNOS) expression in the islets from each treatment group (Figure 5A). The mean cross-sectional islet area was greater in db/db mice than lean mice and this was further increased following concomitant HA+SM therapy (Figure 5B); therefore the mean number of F4/80+ and iNOS+ cells was expressed per unit islet area. Co-administration of HA+SM accentuated the reduction in macrophage infiltration that was observed with HA therapy alone (Figure 5C). Furthermore, the number of iNOS^+^ islet cells in HA+SM-treated db/db mice was reduced to a comparable level to that observed in lean mice (Figure 5D).

### HA Down-regulates the Pro-inflammatory Response to Cytokines in a β-Cell Line Independently of HO Activity

**Figure 6:**
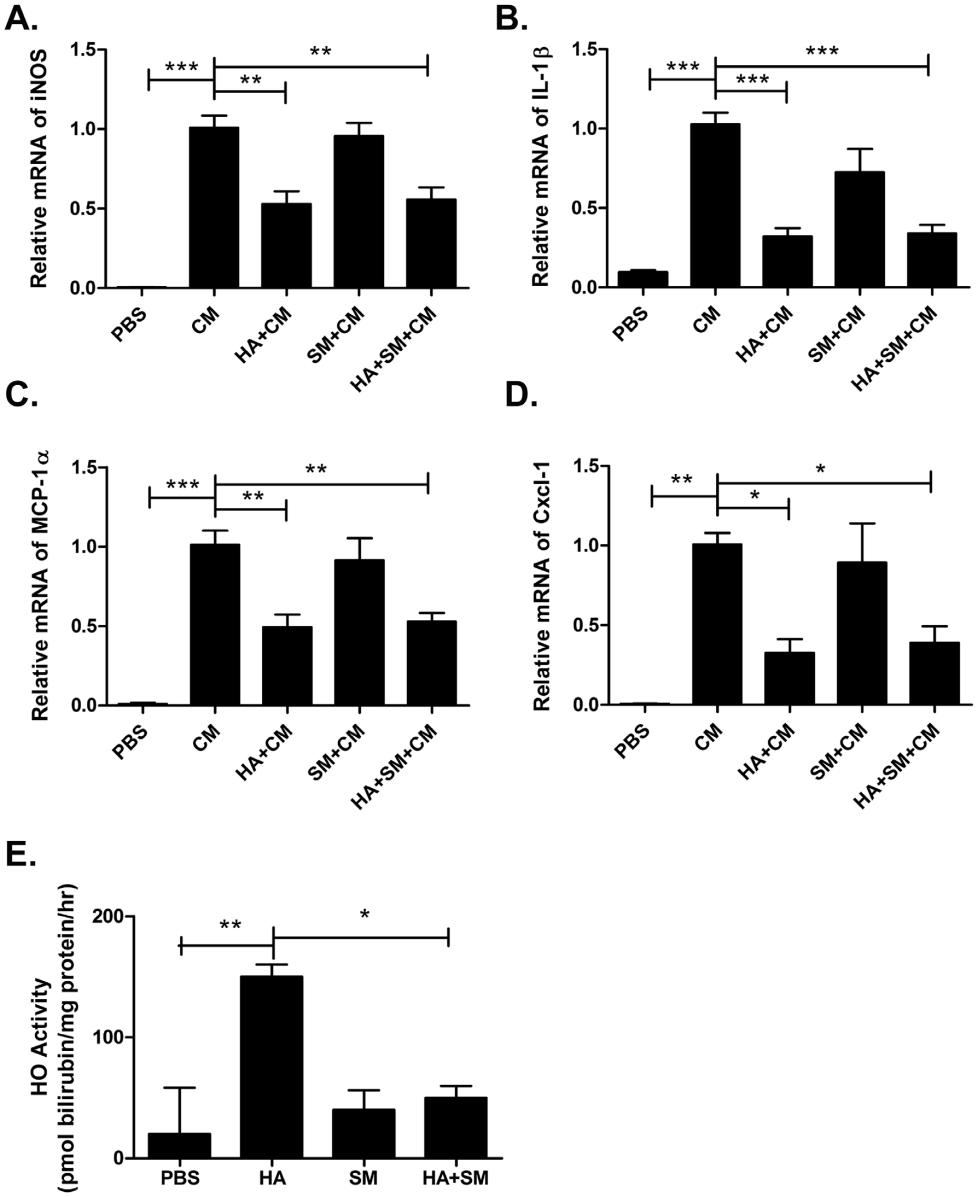
HA±SM reduces cytokine mix (CM) induced pro-inflammatory response in MIN6 β-cell line. Relative gene expression of **A.** iNOS, **B.** IL-1β, C. MCP-1α, **D.** Cxcl-1 in MIN6 β-cells pre-treated with either PBS, HA, SM or HA+SM prior to stimulation with cytokine mix. **E.** HO activity in MIN6 β-cells. *P<0.05, **P<0.01, ***P<0.001. P-values derived from Bonferroni’s multiple comparison post-test when P<0.05 by one-way analysis of variance. The data are means ± s.e.m from n = 4 independent experiments/group.

The anti-inflammatory potential of HA in β-cells was further assessed in an *in vitro* model employing stimulation of the β-cell (MIN6) line with a pro-inflammatory cytokine mix (CM). Administration of HA resulted in a significant reduction in the expression of the iNOS, IL-1β, MCP-1α and Cxcl-1 genes in MIN6 cells in response to stimulation with CM (Figure 6A–D). Pre-treatment with SM abrogated the HA-mediated increase in HO activity in MIN6 cells (Figure 6E), but failed to reverse the anti-inflammatory effects of HA (Figure 6A–D).

### The Severity of Hyperglycaemia was Inversely Correlated to Hepatic Heme Concentration

**Figure 7:**
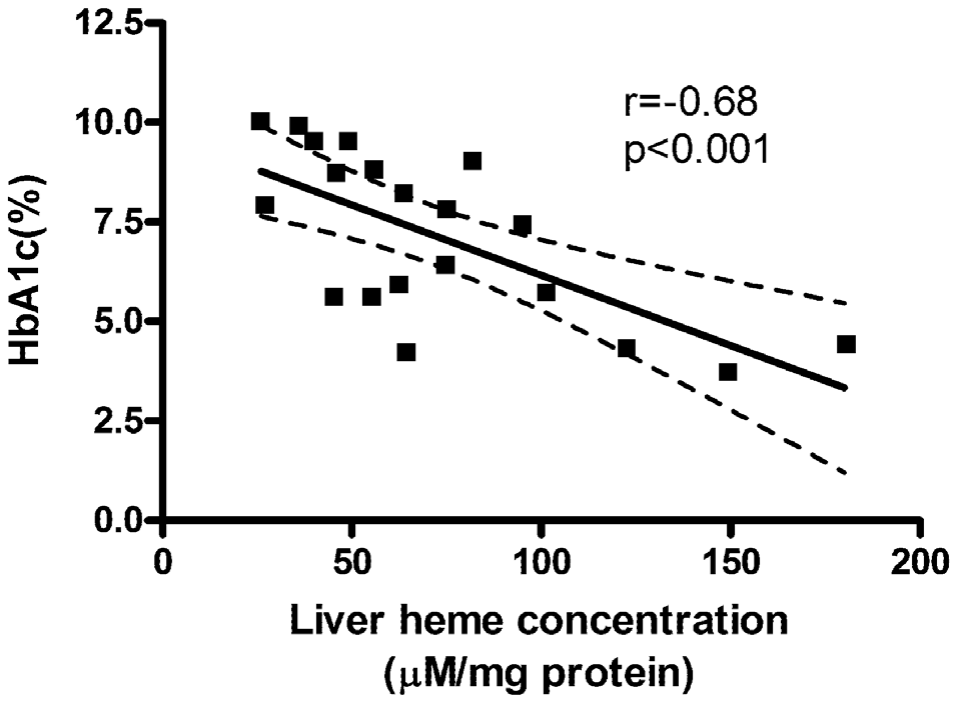
Graph of the correlation between hepatic heme concentration and terminal level of glycosylated haemoglobin (HbA_1c_).

Concurrent administration of HA+SM resulted in a marked accumulation of heme in the liver of db/db mice compared with their PBS-treated counterparts (Table 2). Furthermore, the hepatic heme concentration was inversely correlated to HbA1c levels (Figure 7), suggesting that higher levels of heme were associated with improved glycaemic control.

## Discussion

In the current study, we determined that the heme component of HA ameliorates hyperglycaemia in the db/db mouse model of type 2 diabetes; however, in contrast to previous research [3], we observed that concomitant administration of an inhibitor of HO activity, SM, does not abrogate the anti-diabetic effect of HA but rather accentuates it, indicating that the primary mode of action of heme is not via an increase in HO activity.

Although heme has previously been shown to ameliorate experimental type 2 diabetes, it was administered in the form of intraperitoneal injections of hemin [3]–[5], an approach that is impractical for use in humans. As a prelude to a clinical trial of heme therapy for type 2 diabetes, we administered HA to db/db mice to determine the efficacy of a more soluble and translationally relevant preparation which is currently licensed in Europe for the treatment of acute porphyria. Encouragingly, serial intravenous injections of HA resulted in a modest reduction of blood glucose levels in db/db mice.

We hypothesised that HA was acting via an increase in HO activity, as has been demonstrated previously with hemin [3]; however, despite restoring HO activity to baseline levels, concurrent administration of SM augmented the favourable metabolic effects of HA therapy. We acknowledge that the assessment of HO activity is based solely on hepatic levels and given the key phenotypic changes observed in adipose tissue, we attempted to also confirm this in epididymal fat pads, however this was unsuccessful for technical reasons. However, SM did successfully inhibit HO activity in the 3T3-L1 pre-adipocyte cell line, therefore we have no reason to suspect that a similar effect was not observed in adipose tissue *in vivo*. The reason for the discrepancies between the effects we observed with concurrent administration of heme and inhibition of HO activity and those of previous studies are unclear. We employed an identical regime of SM administration to that used previously in abrogating an increase in HO activity in ob/ob mice [7]. While it is possible that SM may have specific off-target effects, administration of CrMP, an alternative inhibitor of HO activity that was employed in previous studies to suppress the hemin-mediated increase in HO activity [3] had an identical effect to SM on adipogenesis *in vitro*. That the adipogenic potential of HA was greater in vascular stromal fraction cells from HO-1^+/−^ mice compared with WT mice, supports the concept that the metabolic effects of HA are accentuated rather than diminished by a reduction in HO activity and that therefore induction of HO activity is not the primary mechanism for the anti-diabetic effect of heme. This is in keeping with recent studies in which mice with myeloid-specific HO-1 haploinsufficiency were protected from diet-induced insulin resistance [14].

A critical question is how HA and SM interact to produce such a profound reduction in blood glucose levels in db/db mice. One possibility is that concurrent administration of SM potentiates the rise in intracellular heme concentration achieved by HA alone by inhibiting activity of HO, the predominant heme degrading enzyme. This is supported by the close inverse correlation between the hepatic heme concentration and glycosylated haemoglobin levels. That increased heme may ameliorate hyperglycaemia in db/db mice is of particular interest given recent data indicating that heme is an endogenous ligand for the nuclear hormone receptor, Rev-erb-α. Heme binding to Rev-erb-α reduces gluconeogenesis in hepatocytes *in vitro*, and Rev-erb agonists ameliorate glucose intolerance in mice with diet-induced obesity [8].

It is evident that co-administration of HA and SM had pleotropic effects on several organs that are integral to glucose homeostasis. Administration of SM potentiated the anti-diabetic effect of HA despite promoting further weight gain and increasing adiposity. The expansion of adipose tissue was driven by hyperplasia rather than hypertrophy and mediated at least in part by differentiation of pre-adipocytes into adipocytes as reflected by the reduction in expression of the pre-adipocyte gene, Pref-1 and the increase in PPAR-γ, a gene highly expressed in differentiated adipocytes [15], [16]. In addition, the marked reduction in fasting non-esterified fatty acid (NEFA) levels attained with HA+SM therapy suggests that concomitant therapy may also inhibit lipolysis. Despite the increase in adiposity, the combination of HA+SM promoted a metabolically favourable adipose tissue phenotype. While leptin levels increased markedly with HA+SM treatment, as would be expected with the increase in adiposity, serum adiponectin concentration was restored to the level observed in lean mice. This may promote insulin sensitivity via stimulating AMPK activity in muscles [17]. Indeed, the serial HOMA–IR data point to an early reduction in insulin resistance and this may be an integral component of the anti-diabetic effect of combinatorial treatment. In keeping with previous studies (3–5), HA+SM therapy may have selective anti-inflammatory properties in adipose tissue. HA+SM reduced gene expression of the chemotactant MCP-1, which may account for the reduced infiltration of activated macrophages (CD68 and iNOS genes). These results must be treated with caution, however, given the fact that there was no difference between treatment groups in TNF-α gene expression or F4/80 staining.

The adipogenic potential of HA and SM is supported by the *in vitro* data where co-administration of SM potentiated the ability of HA to promote differentiation of the pre-adipocyte 3T3-L1 cell line into adipocytes. This was associated with an increase in intracellular heme concentration, which is pertinent as heme has previously been shown to promote adipogenesis *in vitro*, as is also observed with synthetic Rev-erb-α ligands [18]. Interestingly, cobalt protoporphyrin also promoted adipogenesis, suggesting that this may be a generic feature of porphyrins, rather than specific to heme *per se*.

Progressive β-cell dysfunction is a key factor in the pathogenesis of type 2 diabetes in humans and this is replicated in db/db mice as evidenced by the progressive fall in the HOMA-β in the PBS-treated group during the *in vivo* study. Concomitant treatment with HA and SM partially reversed the fall in the HOMA-β in the later stages of the study. Islet inflammation, and in particular macrophage accumulation, plays a key role in β-cell dysfunction in type 2 diabetes [12], [19], therefore it is pertinent that administration of HA and SM not only dampened the inflammatory response to cytokines in the MIN6 β-cell line, but also reduced macrophage accumulation in the islets of db/db mice. The increased iNOS staining observed in the islets of PBS-treated db/db mice may also contribute to β-cell dysfunction as iNOS-derived nitric oxide inhibits insulin secretion from β-cells *in vitro* [20] and, furthermore, iNOS inhibitors ameliorate β-cell dysfunction in rodent models of type 2 diabetes [13]. Hence, the marked reduction in islet iNOS staining observed with concomitant HA and SM treatment may be a key mechanism for the preservation of β-cell function observed. Furthermore, the Rev-erb-α agonistic properties of heme may also be important, as it has recently been demonstrated that Rev-erb-α promotes insulin exocytosis and β-cell proliferation in MIN6 β-cells *in vitro* [21].

In conclusion, the present study confirms the anti-diabetic efficacy of HA in experimental type 2 diabetes via pleiotropic effects on adipose tissue and by reducing islet inflammation. These effects were potentiated by inhibition of HO activity; hence the present study challenges an established paradigm that porphyrin-based compounds ameliorate type 2 diabetes via induction of HO activity. Future studies are required to establish the anti-diabetic efficacy of HA in the diet-induced model of type 2 diabetes and to incisively delineate the molecular mechanisms responsible for pleotropic effects of heme on metabolic processes *in vivo*.
